# Adjuvant chemotherapy *vs* radiotherapy in high-risk endometrial carcinoma: results of a randomised trial

**DOI:** 10.1038/sj.bjc.6603279

**Published:** 2006-07-25

**Authors:** R Maggi, A Lissoni, F Spina, M Melpignano, P Zola, G Favalli, A Colombo, R Fossati

**Affiliations:** 1 Clinica ‘L. Mangiagalli’, Università degli Studi di Milano, Milano, Italy; 2 Ospedale ‘San Gerardo’, Università degli Studi Milano ‘Bicocca’, Monza, Italy; 3 Azienda Ospedaliero-Universitaria di Parma, Università degli Studi di Parma, Italy; 4 Ospedale Mauriziano ‘Umberto I’, Università degli Studi di Torino, Italy; 5 Ospedali Civili di Brescia, Università degli Studi di Brescia, Italy; 6 Ospedale ‘A. Manzoni’, Lecco, Italy; 7 Department of Oncology, Istituto ‘Mario Negri’, Via Eritrea 62, 20157 Milano, Italy

**Keywords:** endometrial cancer, adjuvant therapy, randomised clinical trial, radiotherapy, chemotherapy

## Abstract

Patients with high-risk endometrial carcinoma (stage IcG3, IIG3 with myometrial invasion >50%, and III) receive adjuvant therapy after surgery but it is not clear whether radiotherapy (RT) or chemotherapy (CT) is better. We randomly assigned 345 patients with high-risk endometrial carcinoma to adjuvant CT (cisplatin (50 mg m^−2^), doxorubicin (45 mg m^−2^), cyclophosphamide (600 mg m^−2^) every 28 days for five cycles, or external RT (45–50 Gy on a 5 days week^−1^ schedule). The primary end points were overall and progression-free survival. After a median follow-up of 95.5 months women in the CT group as compared with the RT group, had a no significant hazard ratio (HR) for death of 0.95 (95% confidence interval (CI), 0.66–1.36; *P*=0.77) and a nonsignificant HR for event of 0.88 (95% CI, 0.63–1.23; *P*=0.45). The 3, 5 and 7-year overall survivals were 78, 69 and 62% in the RT group and 76, 66 and 62% in the CT group. The 3, 5 and 7-year progression-free survivals were, respectively, 69, 63 and 56 and 68, 63 and 60%. Radiotherapy delayed local relapses and CT delayed metastases but these trends did not achieve statistical significance. Overall, both treatments were well tolerated. This trial failed to show any improvement in survival of patients treated with CT or the standard adjuvant radiation therapy. Randomised trials of pelvic RT combined with adjuvant cytotoxic therapy compared with RT alone are eagerly awaited.

Endometrial carcinoma is the most common cancer of the female genital tract; it accounts for 7% of all cancers in women and has an incidence of 20/100 000 women year^−1^ in developed countries (Pisani *et al*, 2002). The tumour is diagnosed in 75–80% of cases at FIGO stage 1, which has 5-year survival rates of 80–90%. The cornerstone of treatment is surgery (Look, 2002). In the early stages, brachytherapy or external radiation therapy is advocated by some as the optimal adjuvant treatment, although recent randomised studies have shown that pelvic radiotherapy (RT) improves loco regional control but not overall survival in patients with low risk (Creutzberg *et al*, 2000) and intermediate risk cancers (Keys *et al*, 2004). Adjuvant therapy is considered a mainstay of management for advanced disease but the best therapy to prevent recurrence is still controversial. Radiation therapy, either external alone or combined with vaginal brachytherapy, seems to lower the incidence of local recurrence, but has a questionable role in preventing tumour spread, and distant metastases remain a significant cause of failure in these patients. Protocols for adjuvant systemic chemotherapies have therefore been developed with a view to preventing distant recurrence.

Regimens active in advanced endometrial cancer usually include a combination of an anthracycline compound and cisplatin, with or without an alkylating agent. Cyclophosphamide, doxorubicin (adriamycin) and cisplatin (CAP regimen) have given objective responses in up to 76% of patients with advanced or recurrent endometrial carcinoma (Turbow *et al*, 1985; Hancock *et al*, 1986; Burke *et al*, 1991; Dunton *et al*, 1991) although the role of cyclophosphamide in combination regimens remains controversial (Thigpen *et al*, 1985).

The aim of this randomised study was to assess whether adjuvant chemotherapy (CT) confers an advantage for overall and progression-free survival and on the incidence of local and distant relapses over standard pelvic RT, in high-risk patients without residual tumour

## PATIENTS AND METHODS

### Eligibility and exclusion criteria

To be eligible for this study, patients had to have histologically confirmed endometrioid, adenoacanthoma or adenosquamous carcinoma and FIGO stage Ic G3, IIa-bG3 with deep myometrial invasion (50% or more) or stage III disease. To rule out FIGO stage IV disease all patients underwent chest radiography and abdominal–pelvic ultrasound. Women had to have had surgery as primary treatment and no previous neoadjuvant therapy or adjuvant brachytherapy. Informed consent fulfilling the requirements of local human biomedical ethics committees was obtained from all patients before entry into the study.

Patients were excluded for any of the following criteria: Karnofsky performance status less than 80; clear-cell, serous papilliferous carcinoma or undifferentiated carcinoma; a second malignant disease (with the exception of surgically treated *in situ* carcinoma of the uterine cervix or basal cell carcinoma of the skin); serious cardiopathy that would contraindicate the use of adriamycin; inadequate liver or renal function (bilirubin levels >1.5 mg dl^−1^ or a serum creatinine >1.5 mg dl^−1^); inadequate bone marrow function, defined as a leukocyte count <4 × 10^9^ l^−1^ and/or platelet count <100 × 10^9^ l^−1^; macroscopic residual tumour.

### Treatment schedules

All patients underwent surgery as primary treatment and with no restrictions on the extent of primary surgery. The standard surgical treatment was total abdominal hysterectomy with bilateral salpingo-oophorectomy (TAH-BSO) with or without partial colpectomy (excision of the vaginal cuff/upper third of vagina) and selective pelvic and lumbo-aortic node sampling. The other surgical procedures were vaginal hysterectomy with BSO, and radical hysterectomy.

After surgical staging and histological evaluation the patients were allocated to one of the two treatments, as follows:

Chemotherapy had to start within 30 days from surgery. Cyclophosphamide 600 mg m^−2^, adriamycin 45 mg m^−2^ and cisplatin 50 mg m^−2^ (CAP regimen) were administered every 28 days for five cycles. In case of bone marrow suppression the doses were adjusted following these criteria: leukocyte count 2.5–3.0 × 10^9^ l^−1^ or platelet count <100 × 10^9^ l^−1^ was managed by a 50% dose reduction of cyclophosphamide and adriamycin; in case of leukocyte count <2.5 × 10^9^ l^−1^ the CT was interrupted until the toxicity completely resolved. In case of severe nephrotoxicity, defined as creatinine clearance ⩽40 ml min^−1^ per 1.73 m^2^, cisplatin was interrupted and therapy continued with adriamycin and cyclophosphamide only. For patients who experienced hepatic dysfunction the protocol stipulated a 50% and a 75% reduction in the adriamycin dosage if bilirubin was between 1.5 and 3 mg dl^−1^, and if it was more than 3 mg dl^−1^.

Radiotherapy had to start within 30 days after surgery. External radiation therapy was adopted for a total of 45–50 Gy in 5–7 weeks (1.7–2 Gy day^−1^ × 5 days week^−1^). The upper limit of the pelvic field was at L5, the lower limit at the lower limit of the ischial tuberosity, and the lateral limits fell behind the border of the lateral and common iliac lymph nodes. Patients who had lymph node involvement received additional lumbo-aortic lymph node irradiation with the upper limit at L1, with 45 Gy in 5–7 weeks (1.5–1.8 Gy day^−1^ × 5 days week^−1^).

### Follow-up

The patients were evaluated with periodic visits every 3 months in the first 3 years, every 6 months in the next 2 years, then annually. At each follow-up, symptoms were recorded, and abdominal palpation and pelvic examination were performed. Vault smears were taken every 6 months for the first 2 years, then annually. Chest radiograms were taken once a year. Other diagnostic tests were carried out whenever a recurrence was suspected. Any complications were assessed at each visit. Chemotherapy toxicities were graded according to World Health Organization criteria (World Health Organization, 1979).

### Statistical methods

The primary end points for this trial were overall survival, defined as the time from randomisation to death, irrespective of the cause, and progression-free survival, defined as the time from randomisation to the earliest tumour relapse, or death. We calculated that a total of 345 patients would permit the detection of a true 35% relative decrease in the mortality hazard rate of patients in the CT arm, with an 80% chance, when the type-I error is limited to 0.05 (two tails). Assuming, for example, a 5-year overall survival of 60% for the RT group, a hazard ratio (HR) of 0.65 corresponds to an absolute increase of 12% in 5-year overall survival.

Randomisation was carried out centrally by telephone at the L Mangiagalli Institute (Milan, Italy) and patients were stratified by institution and stage of disease. Overall and progression-free survival curves were constructed with the Kaplan–Meier method.

The cumulative incidences of local (central pelvic, including vaginal cuff recurrence, lateral pelvic and vaginal) and distant relapses at a time point were calculated using the Kaplan–Meier approach accounting for the presence of competing risk events. Competing risk events were considered distant relapse or death without relapse when calculating the cumulative incidence of local relapse and local relapse or death without relapse when calculating the cumulative incidence of distant relapse (Satagopan *et al*, 2004). We considered the incidence of local and distant relapses as first failure only. If local and distant relapses were detected concurrently these cases were shown on the cumulative incidence curves of both local and distant relapses.

The duration of follow-up was calculated using the Kaplan–Meier estimate of the median duration by reversing the status of censoring and death in the data set. The log-rank test and Cox proportional hazards regression models were used to compare time-to-event distributions between the treatment groups in univariate and multivariate analyses. The 95% CIs for the HRs of treatment effect and other prognostic factors were provided to indicate the range of values consistent with the observed data and were determined from the asymptotic s.e. in the Cox regression model. All statistical tests were two-sided. The analysis was by intention-to-treat and patients were analysed according to the assigned treatment.

The results are reported according to the revised CONSORT statement (Moher *et al*, 2001).

## RESULTS

### Patients characteristics and compliance with treatment

**Figure 1:**
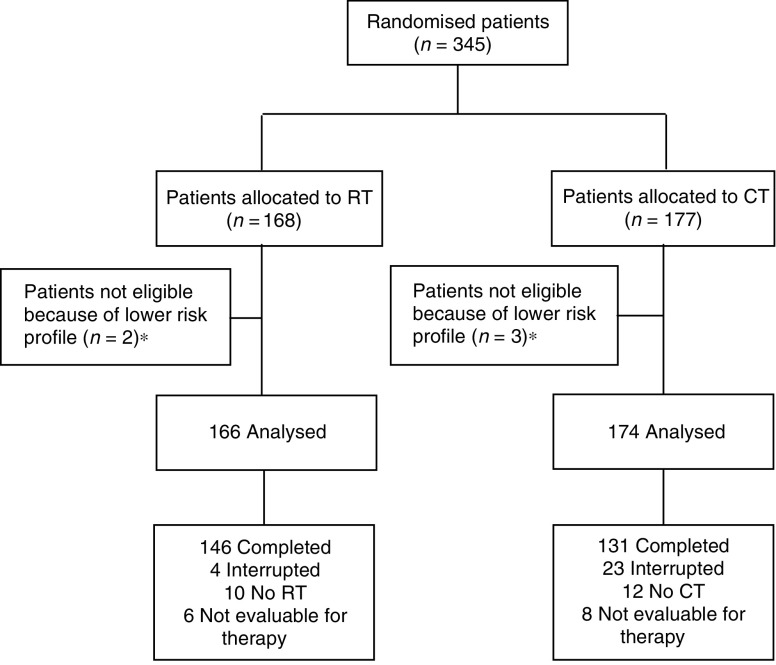
Flow chart of the progress of patients through the trial (Adapted from Begg C, Cho M, Eastwood S, *et al.* proving the quality of reporting of randomised controlled trials: the CONSORT statement. JAMA 1996;276;637–639). ^*^ Lower risk profile=FIGO stage IaG1-3, IbG1-3, IcG1-2, IIaG1-2, IIbG1-2.

Between January 1, 1990 and December 31, 1997, 491 patients with high-risk endometrial carcinoma were consecutively referred to 29 institutions throughout Italy. A total of 345 patients were deemed eligible for this study, with 168 randomly assigned external RT and 177 adjuvant CT (Figure 1).

Table 1 lists the distribution by treatment group of patients according to age, FIGO stage, grading, degree of myometrial invasion and type of primary surgery. The two groups were similar across all categories. About one third of the patients had FIGO stage I–II disease and two thirds stage III, and approximately 70% of the patients had myometrial invasion deeper than 50%. Of the 340 patients analysed, 315 (93%) underwent TAH-BSO.

Of the 166 patients assigned RT, 146 (88%) completed treatment as planned (Figure 1); only four patients (2%) stopped treatment because of toxicity (investigators were not required to report the specific toxic effect that prompted treatment cessation); 10 patients (6%) declined treatment. Of the 174 patients assigned CT, 131 (75%) received five treatment cycles as planned and 154 (89%) received at least one cycle and were assessable for toxicity (six, four, four and nine patients received only one, two, three and four courses of CAP, respectively, mainly because of excessive bone marrow toxicity). In all, 12 patients (7%) declined adjuvant CT.

### Toxicity

We had toxicity data for 146 (97%) of the 150 patients who started RT (RT). Major late toxic effects were gastrointestinal, including five cases of bowel obstruction with three of these patients requiring surgical intervention, six cases of grade 3 radiation proctitis, and 13 reports of grade 3 diarrhoea (24 patients, 16%). Urinary tract complications (severe actinic cystitis) were recorded in seven patients (5%).

We collected full details about the toxicity of CAP for 123 patients (80% of the 154 patients who had at least one course). Grades 2, 3 and 4 neutropenia occurred in 22 (18%), 38 (31%) and 5 (4%) patients, respectively; 36 patients (29%) had grade 2 anaemia, 5 (4%) had grade 3 anaemia; grade 2 and 3 thrombocytopenia was reported in five (4%) and two patients (2%), respectively. The incidence of nausea and vomiting was relatively low (grade 2 and 3 was reported for 29 (24%) and 12 (10%) patients, respectively, grade 4 for one patient). Other serious toxicities (grade 3) occurred in <3 % of the patients randomised to CT. There were no treatment-related deaths.

### Recurrence and survival

**Figure 2:**
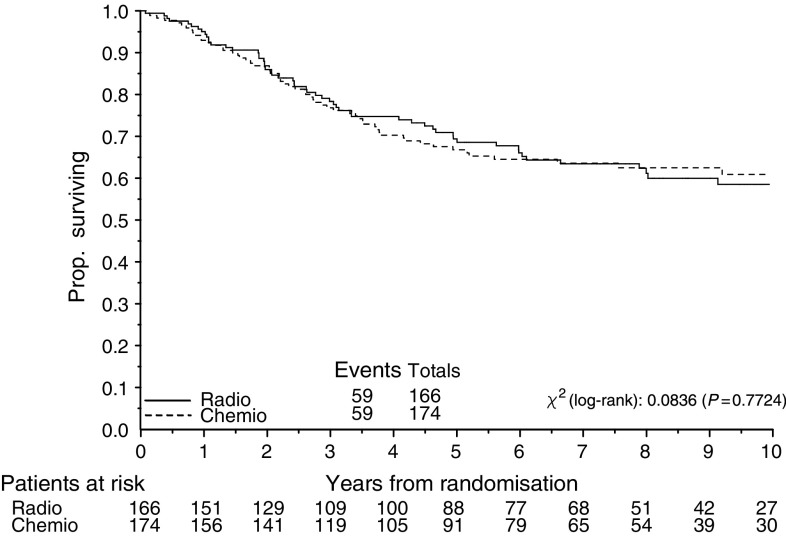
Overall survival of patients with high-risk endometrial carcinoma (stage IcG3, IIG3 with myometrial invasion >50%, and III) receiving adjuvant radiotherapy (Radio) or chemotherapy (Chemio). Five-year overall survival was 69% and 66% respectively for adjuvant radiotherapy and chemotherapy.

**Figure 3:**
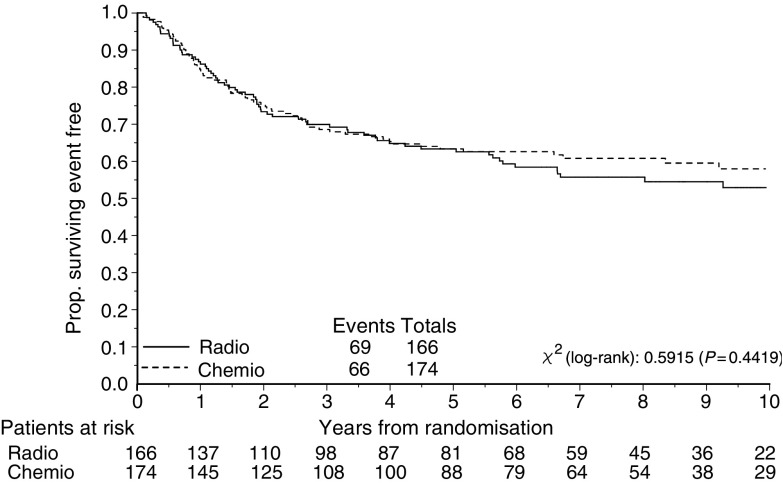
Progression-free survival of patients with high-risk endometrial carcinoma (stage IcG3, IIG3 with myometrial invasion >50%, and III) receiving adjuvant radiotherapy (Radio) or chemotherapy (Chemio). Five-year progression-free survival was 63% and 63%.

At the median follow-up time of 95.5 months (interquartile range 62 to 122 months), 135 events (recurrences or deaths, whichever came first) had occurred among the 340 randomised patients: 60 recurrences and nine deaths as first event of the 166 patients on RT, and 56 recurrences and 10 deaths as first event of the 174 patients on CT. The overall number of observed deaths was 118 (35%), 59 in the RT arm and 59 in the CT arm. Comparison of the Kaplan–Meier curves for death (Figure 2) or first event (Figure 3) gave nonsignificant HRs of 0.95 (CI=0.66–1.36, *P*=0.78) and 0.88 (CI=0.63–1.23, *P*=0.45), respectively (Figure 2).

The overall survival of the patients on CT was 76% (CI=70–83%), 66% (CI=59–73%) and 62% (CI=55–70%) at the third, fifth and seventh year, respectively, and 78% (CI=71–84%), 69% (CI=61–76%) and 62% (CI=54–71%) for patients on RT at the same time points. The progression-free survival of the patients on CT was 68% (CI=61–75%), 63% (CI=55–70%) and 60% (CI=52–67%) at the third, fifth and seventh year, respectively, and 69% (CI=62–77%), 63% (CI=55–70%) and 56% (CI=46–63%) for patients on RT.

**Figure 4:**
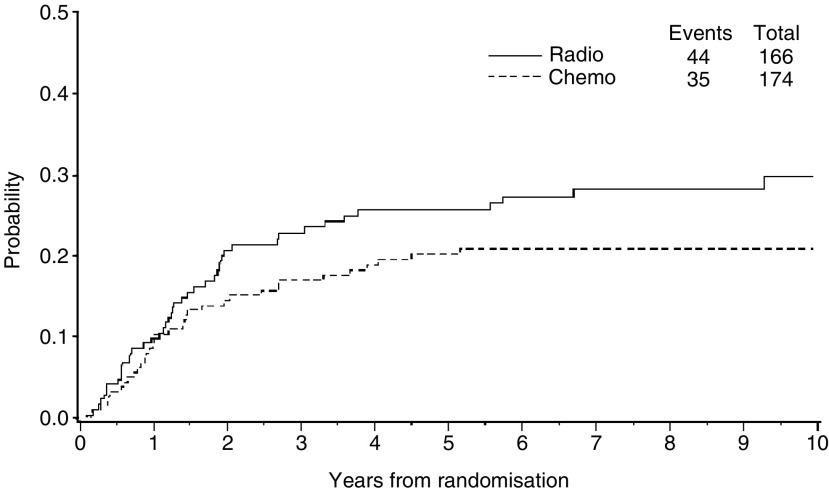
Cumulative incidence of distant relapses for patients with high-risk endometrial carcinoma (stage IcG3, IIG3 with myometrial invasion >50%, and III) receiving adjuvant radiotherapy (Radio) or chemotherapy (Chemo).

**Figure 5:**
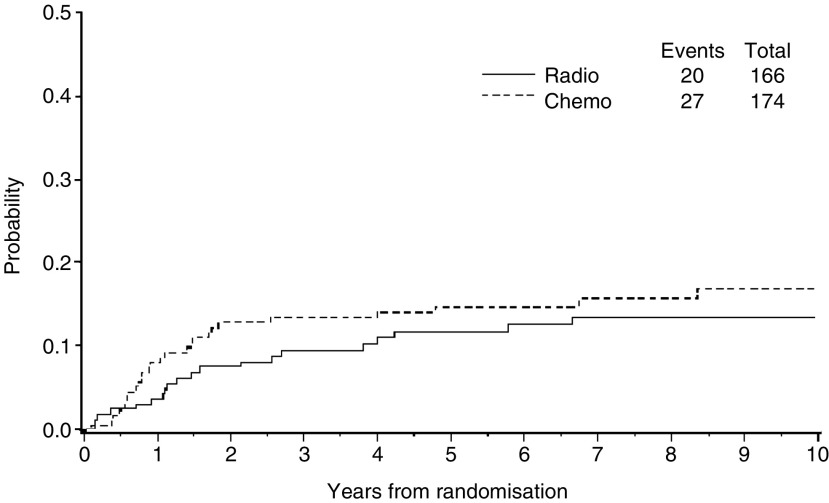
Cumulative incidence of local (central pelvic, including vaginal cuff recurrence, lateral pelvic and vaginal) relapses for patients with high-risk endometrial carcinoma (stage IcG3, IIG3 with myometrial invasion >50%, and III) receiving adjuvant radiotherapy (Radio) or chemotherapy (Chemo).

In the multivariate proportional hazard model (Table 2) age, grading, depth of myometrial invasion and FIGO stage were all significantly associated with progression-free and overall survival. Multivariate analysis confirmed there was no real difference between CT and RT in progression-free and overall survival. Figures 4 and 5 depict the cumulative incidence, after adjusting for competing risks, of distant or local relapses by treatment arm. Among the 166 patients randomised to RT, the initial site of recurrence was distant (extra-abdominal or liver) in 35 (21%), local in 11 (7%), concurrent distant and local in nine (5%), and of unknown type in five (3%). Among the 174 patients randomised to CT, the initial site of recurrence was distant in 27 (16%), local in 19 (11%), concurrent local and distant in eight (5%), and of unknown type in two (1%). Although this study was not powered to detect clinically significant differences in the incidence of relapses, CT seemed to prevent or delay distance relapses more than RT (Figure 4) while the RT seemed to prevent or delay local relapses in comparison with CT (Figure 5).

## DISCUSSION

To our knowledge, this was the first trial to randomise patients with high-risk endometrial cancer to compare the efficacy of adjuvant CT and standard pelvic RT. This trial failed to show an improvement in progression-free and overall survival in patients treated with one or the other treatment protocol. Both therapeutic approaches were associated with acceptable toxicities.

Optimal adjuvant therapy for patients with high-risk disease is poorly defined. Three randomised clinical trials comparing adjuvant pelvic RT with brachytherapy (Aalders *et al*, 1980) or observation (Creutzberg *et al*, 2000; Keys *et al*, 2004) all showed a highly significant reduction of the risk of loco regional relapse with pelvic irradiation but no clear trend toward prevention of distant metastases or improvement of overall survival (Creutzberg, 2004).

Evidence on the effects of platinum-based systemic CT in an adjuvant setting for endometrial cancer come from prospective nonrandomised small series of patients. Therefore, comparisons of adjuvant CT with adjuvant radiation were largely indirect and tended to favour CT (Aoki *et al*, 2001, 2004) although it was clear that CT did not prevent loco-regional recurrences. Our trial directly compared adjuvant pelvic RT and CT, with an experimental design similar to the GOG-122 trial that has been recently published (Randall *et al*, 2006). The GOG study randomised women with stage III–IV endometrial carcinoma to whole abdominal irradiation or platinum-doxorubicin CT and reported a clear benefit of combined cytotoxic therapy in terms of progression-free survival and overall survival. The HRs of progressing and dying relative to RT adjusted for stage were 0.71 (95% CI=0.55–0.91) and 0.68 (95% CI=0.52–0.89), respectively. Our data do not agree with these results but there are important differences between the two studies that need comment: in the GOG trial a sizeable proportion of patients (about 25%) were reported as having other and more aggressive pathologic types than endometroid or adenosquamous (ie serous and clear-cell) and included stage IV disease and patients with residual tumour up to 2 cm, so the study population had a much higher risk of relapse and death (5-year overall survival was 42% in the RT arm, 53% in the CT arm); the CT regimen was a doublet (doxorubicin and cisplatin) not the triplet used in our study (doxorubicin, cisplatin and cyclophosphamide); the doxorubicin dose in the GOG protocol was higher and CT was cycled every 3 weeks instead of 4 weeks for seven courses with an additional course of cisplatin, instead of five courses; RT was whole abdominal irradiation with a boost to pelvis, not just limited to the pelvis as in our study; in the GOG trial there were 12 treatment-related deaths (four RT, eight CT).

We thought that the differences between our study and GOG-122 might have been related to differences in histological subtypes, which were more aggressive and possibly more chemoresponsives in the GOG-122 population but a recent pooled analysis of four GOG studies showed similar response rates of endometroid and serous tumours to CT (Scott *et al*, 2005). Thus, differences can only be explained with the combination of the more aggressive GOG-122 CT schedule and a target patient population with poorer prognosis.

When our study was designed, the drug combination proposed in the protocol (the CAP regimen, cycled every 4 weeks) was considered effective in advanced endometrial and ovarian cancer (de Oliveira *et al*, 1990; Burke *et al*, 1991). The CAP regimen, although usually cycled every 3 weeks, is still widely used and many clinicians in Europe use it as first-line treatment for ovarian cancer (International, 2002). The doxorubicin dose in the CAP regimen is lower than the dose used in the GOG-122 trial but a recent retrospective analysis of three phase III trials in 630 patients indicated that the risk of cardiotoxicity (ie irreversible cardiac heart failure) began to increase with cumulative doses of conventional doxorubicin as low as 300 mg m^−2^ (Swain *et al*, 2003). The GOG-122 maximum allowable cumulative dose of doxorubicin was 420 mg m^−2^ and this should set the estimated risk range of cardiomyopathy between 5 and 16%.

The cumulative incidence curves of local and distant relapses in our study suggest that RT might achieve better loco-regional control while systemic CT might control distant metastases better. Although both approaches are still unsatisfactory, since the risk of progression or death remains high, this encouraging evidence of clinical activity suggest they might be used concurrently or sequentially use in an adjuvant setting. Only one randomised trial assessed the efficacy of adjuvant CT after RT compared with RT alone (Morrow *et al*, 1990). This GOG trial randomised high-risk patients to receive doxorubicin or no further therapy after postoperative pelvic RT. No differences emerged in overall survival but the trial was probably underpowered to detect a clinically important effect.

In an attempt to maximise treatment activity several cooperative groups have launched randomised trials comparing RT plus CT with RT alone in high-risk endometrial cancer (ILIADE group, EORTC 55991). These will further define the optimal sequencing of treatment approaches including concomitant RT and CT that showed acceptable safety and toxicity in preliminary analyses (Greven *et al*, 2004).

In this setting, taxane-platin combinations seem promising especially, considering the possible role of taxane compounds as radiation enhancer (Liebmann *et al*, 1994a, 1994b).

## Figures and Tables

**Table 1:** Clinical and tumour characteristics

	**RT (166 patients)**		**CT (174 patients)**	
	**No.**	**%**	**No.**	**%**
Median age (25th–75th percentiles), years	62 (55–67)		63 (57–69)	
				
*FIGO stage*				
Ic	43	26	47	27
IIa	2	1	0	0
IIb	15	9	14	8
IIIa	64	39	73	42
IIIb	5	3	2	1
IIIc	37	22	38	22
				
*Histological grade*				
1	19	12	13	8
2	43	26	59	34
3	98	59	94	54
Missing data	6	4	8	5
				
*Myometrial invasion*				
M0 (no invasion)	1	1	0	0
M1 (invasion⩽50%)	37	22	42	24
M2 (invasion >50%)	118	71	125	72
Missing data	10	6	7	4
				
*Primary surgery*				
Total abdominal hysterectomy+bilateral salpingo-oophorectomy (TAH-BSO)+partial colpectomy	108	65	110	63
TAH-BSO	44	27	53	31
TAH+monolateral salpingo-oophorectomy (MSO)	4	2	0	0
Vaginal hysterectomy-BSO	1	1	2	1
MEIGS radical hysterectomy	5	3	2	1
Missing data	4	2	7	4

*Note*: Clinical stage based on the International Federation of Gynecology and Obstetrics surgical staging system.

**Table 2:** Multivariable Cox proportional hazards analysis for progression-free and overall survival

	**Progression-free survival**		**Overall survival**	
	**HR (95%CI)**	***P***	**HR (95%CI)**	***P***
*Treatment arm*				
Radiotherapy^a^	1	0.64	1	0.85
Chemotherapy	0.92 (0.65–1.30)		1.04 (0.72–1.50)	
				
*Age (years)*				
<70^a^	1	0.009	1	0.001
≥70	1.71 (1.15–2.56)		1.99 (1.31–3.02)	
				
*Tumor grade*				
1 or 2^a^	1	<0.0001	1	<0.0001
3	2.74 (1.78–4.22)		3.09 (1.96–4.89)	
				
*Myometrial invasion*				
⩽50%^a^	1	0.001	1	0.002
>50%	2.20 (1.36–3.57)		2.22 (1.34–3.69)	
				
*Stage*				
I or II^a^	1	<0.0001	1	<0.0001
III	2.70 (1.75–4.15)		3.17 (2.00–5.01)	

HR=hazard ratio, CI=confidence interval

a Reference category

**Table A1:** 

Ospedale ‘S Gerardo’, Università degli Studi Milano Bicocca, Monza	A Gabriele, G Caspani, M Signorelli, S Corso
Ospedale ‘S Raffaele’, Milano	G Mangili, E Rabaiotti, E Garavaglia
Ospedale ‘S Matteo’, Università degli studi di Pavia, Pavia	S Tateo
Ospedale ‘Valduce’, Como	L Redaelli, R Colleoni
Ospedali Civili di Brescia, Università degli Studi di Brescia, Brecia	E Sartori, A Gambino, G Tognon, F Ramazzotto, G Spinetti
Ospedale ‘S Antonio Abate’, Gallarate	C Borsani, G Ranchet
Ospedale di Circolo ‘Fondazione Macchi’, Università degli Studi dell’Insubria, Varese	PF Bolis, N Donadello, C Apolloni
Ospedale ‘S Paolo’, Università degli Studi di Milano, Milano	D Perugino
I Clinica Ostetrico Ginecologica, Clinica ‘L Mangiagalli’, Università degli Studi di Milano, Milano	G Scarfone
Policlinico ‘Gemelli’, Università Cattolica del Sacro Cuore, Roma	G Scambia, S Mancuso, PL Benedetti Panici
Arcispedale ‘S Anna’, Ferrara	R Martinello
Ospedale Mauriziano ‘Umberto I’, Torino	ME Jacomuzzi
Ospedale ‘S Maria delle Stelle’, Melzo	A.Uderzo, A.Olivari
II Clinica Ostetrico Ginecologica, Clinica ‘L.Mangiagalli’, Università degli Studi di Milano, Milano	C Restelli, L Cucchi, F Maggi, L Carlini
Ospedale Civile di Voghera, Voghera	C Scarabelli, M Presti, C Beccaria
Ospedale Civile di Desio, Desio	S Arienti
Ospedale Maggiore di Lodi, Lodi	S Garutti
Ginecologia Oncologica – Ospedale ‘S Anna’, Università degli Studi di Torino, Torino	D Katsaros, A Durando, R Bellino
Ginecologia Divisione A – Ospedale ‘S Anna’, Università degli Studi di Torino, Torino	S Danese
Ospedale Civico di Codogno, Codogno	A Frigoli, N Spolti
Azienda Ospedaliera della Valtellina e della Valchiavenna, Sondrio	F Dolci, A Nozza
Azienda Ospedaliera Ospedale di Lecco	N Natale
Azienda Ospedaliera ‘Istituti Ospitalieri di Cremona’, Cremona	C Fertonani
Azienda Ospedaliera Ospedale di Parma, Università degli Studi di Parma, Parma	M Melpignano, C Merisio, R Berretta, E Vadora
Istituto Nazionale dei Tumori ‘Regina Elena’, Roma	M Mariani
Ospedale ‘Cannizzaro’, Catania	P Scollo
Istituto ‘Mario Negri’, Laboratory of Clinical Research in Oncology, Milan	V Torri
	I Floriani
SENDO Foundation, Milan	A Tinazzi

## Appendix A. Study centers and consultants

Table A1

## References

[bib1] Aalders J, Abeler B, Kolstad P, Onsrud M (1980) Postoperative external irradiation and prognostic parameters in stage I endometrial carcinoma. Clinical and histopathologic study of 540 patients. Obstet Gynecol 56: 419–426 6999399

[bib2] Aoki Y, Kase H, Watanabe M, Sato T, Kurata H, Tanaka K (2001) Stage III endometrial cancer: analysis of prognostic factors and failure patterns after adjuvant chemotherapy. Gynecol Oncol 83: 1–5 11585406 10.1006/gyno.2001.6321

[bib3] Aoki Y, Watanabe M, Amikura T, Obata H, Sekine M, Yahata T, Fujita K, Tanaka K (2004) Adjuvant chemotherapy as treatment of high-risk stage I and II endometrial cancer. Gynecol Oncol 94: 333–339 15297170 10.1016/j.ygyno.2004.05.040

[bib4] Burke TW, Stringer CA, Morris M (1991) Prospective treatment of advanced or recurrent endometrial carcinoma with cisplatin, doxorubicin, and cyclophosphamide. Gynecol Oncol 40: 264–267 2013451 10.1016/0090-8258(90)90289-w

[bib5] Creutzberg CL, van Putten WL, Koper PC, Lybeert ML, Jobsen JJ, Warlam-Rodenhuis CC, De Winter KA, Lutgens LC, van den Bergh AC, van de Steen-Banasik E, Beerman H, van Lent M (2000) Surgery and postoperative radiotherapy *vs* surgery alone for patients with stage-1 endometrial carcinoma: multicentre randomised trial. PORTEC Study Group. Post Operative Radiation Therapy in Endometrial Carcinoma. Lancet 355: 1404–1411 10791524 10.1016/s0140-6736(00)02139-5

[bib6] Creutzberg CL (2004) GOG-99: ending the controversy regarding pelvic radiotherapy for endometrial carcinoma? Gynecol Oncol 92: 740–743 14984935 10.1016/j.ygyno.2004.01.009

[bib7] de Oliveira CF, Lacave AJ, Villani C, Wolff JP, di Re F, Namer M, Maskens A, George M, Dalesio O, Rotmensz N (1990) Randomized comparison of cyclophosphamide, doxorubicin and cisplatin (CAP) *vs* cyclophosphamide and doxorubicin (CA) for the treatment of advanced ovarian cancer. A EORTC Gynecological Cancer Cooperative Group study. Eur J Gynecol Oncol 11: 323–330 2097149

[bib8] Dunton CJ, Pfeifer SM, Braitman LE (1991) Treatment of advanced and recurrent endometrial cancer with cisplatin, doxorubicin, and cyclophosphamide. Gynecol Oncol 41: 113–116 2050302 10.1016/0090-8258(91)90268-a

[bib9] Greven K, Winter K, Underhill K, Fontenesci J, Cooper J, Burke T (2004) Preliminary analysis of RTOG 9708: Adjuvant postoperative radiotherapy combined with cisplatin/paclitaxel chemotherapy after surgery for patients with high-risk endometrial cancer. Int J Radiat Oncol Biol Phys 59: 168–173 15093913 10.1016/j.ijrobp.2003.10.019

[bib10] Hancock KC, Freedman RS, Edwards CL (1986) Use of cisplatin, doxorubicin, and cyclophosphamide to treat advanced and recurrent adenocarcinoma of the endometrium. Cancer Treat Rep 70: 789–791 3731140

[bib11] International Collaborative Ovarian Neoplasm group (2002) Paclitaxel plus carboplatin *vs* standard chemotherapy with either single-agent carboplatin or cyclophosphamide, doxorubicin and cisplatin in women with ovarian cancer: the ICON3 randomized trial. Lancet 260: 505–515 10.1016/S0140-6736(02)09738-6 12241653

[bib12] Keys HM, Roberts JA, Brunetto VL, Zaino RJ, Spirtos NM, Bloss JD, Pearlman A, Maiman MA, Bell JG, Gynecologic Oncology Group (2004) A phase III trial of surgery with or without adjunctive external pelvic radiation therapy in intermediate risk endometrial adenocarcinoma: a Gynecologic Oncology Group study. Gynecol Oncol 92: 744–751 14984936 10.1016/j.ygyno.2003.11.048

[bib13] Liebmann J, Cook JA, Fisher J, Teague D, Mitchell JB (1994a) *In vitro* studies of taxol as a radiation sensitizer in human tumor cells. J Natl Cancer Inst 86: 441–446 7907149 10.1093/jnci/86.6.441

[bib14] Liebmann J, Cook JA, Fisher J, Teague D, Mitchell JB (1994b) Changes in radiation survival curve parameters in human tumor and rodent cells exposed to paclitaxel (taxol). Int J Radiat Oncol Biol Phys 29: 559–564 7911794 10.1016/0360-3016(94)90456-1

[bib15] Look K (2002) Stage I endometrial adenocarcinoma evolution of therapeutic paradigms: the role of surgery and adjuvant radiation. Int J Gynecol Cancer 12: 237–249 12060444 10.1046/j.1525-1438.2002.01119.x

[bib16] Moher D, Schulz KF, Altman DG (2001) The CONSORT statement: revised recommendations for improving the quality of reports of parallel-group randomised trials. Lancet 357: 1191–1194 11323066

[bib17] Morrow CP, Bundy BN, Homesley HD, Creasman WT, Hornback NB, Kurman R, Thigpen JT (1990) Doxorubicin as an adjuvant following surgery and radiation therapy in patients with high-risk endometrial carcinoma, stage I and occult stage II: a Gynecologic Oncology Group Study. Gynecol Oncol 36: 166–171 2298404 10.1016/0090-8258(90)90166-i

[bib18] Pisani P, Bray F, Parkin DM (2002) Estimates of the world-wide prevalence of cancer for 25 sites in the adult population. Int J Cancer 97: 72–81 11774246 10.1002/ijc.1571

[bib19] Randall ME, Filiaci VL, Muss H, Spirtos NM, Mannel RS, Fowler J, Thigpen JT, Benda JA, Gynecologic Oncology Group Study (2006) Randomized phase III trial of whole-abdominal irradiation *vs* chemotherapy in advanced endometrial carcinoma: a Gynecologic Oncology Group study. J Clin Oncol 24: 36–44 16330675 10.1200/JCO.2004.00.7617

[bib20] Satagopan JM, Ben-Porat L, Berwick M, Robson M, Kutler D, Auerbach AD (2004) A note on competing risks in survival data analysis. Br J Cancer 91: 1229–1235 15305188 10.1038/sj.bjc.6602102 PMC2410013

[bib21] Scott Mc, Meekin SD, Filiaci VL, Thigpen T, Gallion H, Fleming GF (2005) Importance of histology in advanced and recurrent endometrial cancer patients participating in 1st-line chemotherapy trials: a Gynecologic Oncology Group (GOG) trial. Gynecol Oncol 96: 940

[bib22] Swain SM, Whaley FS, Ewer MS (2003) Congestive heart failure in patients treated with doxorubicin. Cancer 97: 2869–2879 12767102 10.1002/cncr.11407

[bib23] Thigpen JT, Blessing J, DiSaia P (1985) A randomized comparison of Adriamycin with or without Cyclophosphamide in the treatment of advanced or recurrent Endometrial Cancer. Proc Am Soc Clin Oncol 4: 115

[bib24] Turbow MM, Ballon SC, Sikic BI (1985) Cisplatin, doxorubicin, and cyclophosphamide chemotherapy for advanced endometrial Carcinoma. Cancer Treat Rep 69: 465–467 4039978

[bib25] World Health Organization (WHO) (1979) Handbook for Reporting Results of Cancer Treatment. Geneva (Switzerland), Publ No. 48. Out-of-print publication is available online at: http//whqlibdoc.who.int/publications/9241700483.pdf

